# Marked reductions in rates of vancomycin-resistant enterococci (VRE) colonization & disease associated with introduction of a routine hospital-wide bleach cleaning program

**DOI:** 10.1186/1753-6561-5-S6-P24

**Published:** 2011-06-29

**Authors:** ML Grayson, AA Mahony, EA Grabsch, DR Cameron, RD Martin, M Heland, M Petty, S Xie

**Affiliations:** 1 Infectious Diseases, Austin Health, Melbourne, Australia; 2 Medicine, University of Melbourne, Australia; 3 Austin Health, Melbourne, Australia

## Introduction / objectives

To reduce rates of VRE colonization/disease, we introduced a multimodal hospital-wide bleach-based cleaning program (BBCP) that included a new product (sodium hypochlorite 1000ppm + detergent), new standardised (routine and detailed) cleaning practices & modified glove/gown protocols to rely on alcohol-based handrub & sleeveless aprons. Rates of VRE pre- & post-BBCP were compared.

## Methods

Patients in 4 high-risk wards (liver transplant, renal, ICU, hem/oncology) were screened on admission & weekly for rectal VRE colonization, & rates were compared pre-BBCP (Period A [6 mo] – Feb-July 2009) *vs* post-BBCP (Period B1 & B2 – Feb-July & Aug-Jan 2010/11). Rates of VRE bacteremia (per 100 patients blood cultured [100PBC]) & of urinary tract infection [UTI] were compared - Period A *vs* B1 & B2.

## Results

A 37% reduction in newly recognised VRE colonizations was observed post-BBCP (208/1948 patients screened [Period A] *vs* 181/2129 [Period B1] *vs* 143/2141 [Period B2], *p*<0.0001), despite an increase in screening compliance (68.1% *vs* 74.6% *vs* 71.9%, *p*=0.061) and a stable rate of on-admission VRE colonization (38/1461 [2.6%] *vs* 44/1795 [2.5%] *vs* 38/1840 [2.1%], *p*=0.34). VRE bacteremia declined from 0.48/100PBC (14/2935) pre-BBCP to 0.08/100PBC (5/6194) during the 12 mo post-BBCP (*p*=0.0002), with a reduction in UTI cases (24 [A] *vs* 19 [B1] *vs* 17 [B2]).

## Conclusion

The BBCP was associated with a significant reduction in rates of both new VRE colonizations (37% decrease) & VRE disease. This approach potentially represents a new paradigm in the management of VRE.

## Disclosure of interest

None declared.

